# Impact of different chemotherapy regimens on intestinal mucosal injury assessed with bedside ultrasound: a study in 213 AML patients

**DOI:** 10.3389/fonc.2023.1272072

**Published:** 2023-11-01

**Authors:** Edoardo Benedetti, Ginevra Traverso, Giulia Pucci, Riccardo Morganti, Emilia Bramanti, Piero Lippolis, Maria Chiara Susini, Elisa Mazzantini, Rachele Giubbolini, Fabrizio Mavilia, Enrico Capochiani, Emanuele Neri, Chiara Arena, Francesca Cerri, Luigi De Simone, Katia Valentini, Salvatore Massimo Stella, Vittorio Ricchiuto, Benedetto Bruno, Sara Galimberti

**Affiliations:** ^1^ Hematology Operative Unit (UO), Department of Clinical and Experimental Medicine, Azienda Ospedaliero Universitaria Pisana, Pisa, Italy; ^2^ Section of Statistics, Azienda Ospedaliero Universitaria Pisana, Pisa, Italy; ^3^ Institute of Chemistry of Organo Metallic Compounds (ICCOM), Consiglio Nazionale delle Ricerche (CNR), Pisa, Italy; ^4^ General and Peritoneal Surgery Unit, Azienda Ospedaliero Universitaria Pisana, Pisa, Italy; ^5^ Hematology Unit, Azienda USL Toscana Nord Ovest, Livorno, Italy; ^6^ Radiology Unit, Department of Translational Research and New Technologies in Medicine and Surgery, University of Pisa, Pisa, Italy; ^7^ Anesthesia and Maternal-Infantile Resuscitation Unit, Azienda Ospedaliero Universitaria Pisana, Pisa, Italy; ^8^ Italian School of Basic and Emergency Ultrasound (SIUMB), Pisa, Italy; ^9^ Dipartimento di Tecnologie Sanitarie ESTAR, Azienda Ospedaliero-Universitaria Pisana, Pisa, Italy; ^10^ Department of Molecular Biotechnology and Health Sciences, University of Turin, Turin, Italy; ^11^ Hematology Unit, Department of Translational Research and New Technologies in Medicine and Surgery, University of Pisa, Pisa, Italy

**Keywords:** neutropenic enterocolitis, NEC, ultrasound sonography, acute myeloblastic leukemia, chemotherapy

## Abstract

**Introduction:**

Neutropenic enterocolitis (NEC) is a life-threatening complication reported in patients with acute myeloid leukemia (AML) following chemotherapy (CHT). Intensive induction and consolidation CHT may damage intestinal mucosa leading to a NEC episode (NECe). NEC reported mortality may be up to 30-60%. Early US-guided bed-side diagnosis and prompt treatment may substantially improve the survival. An emerging worldwide concern is the intestinal colonization by multi-drug-resistant bacteria especially when patients are exposed to chemotherapy regimens potentially correlated to mucosal damage.

**Methods:**

In our study we prospectively enrolled all AML patients admitted in our leukemia unit to receive intensive induction and consolidation chemotherapy and experiencing chemotherapy-induced-neutropenia (CHTN).

**Results and discussion:**

Overall, we enrolled N=213 patients from 2007 to March 2023. We recorded N=465 CHTN, and N=42 NECe (9.0% incidence). The aim of our study was to assess which chemotherapy regimens are more associated with NEC. We found that ALM1310, followed by 7 + 3 (daunorubicin), 7 + 3 (idarubicin), 5 + 3 + 3 (cytarabine, etoposide, idarubicin), and AML1310 (consolidation) were associated with a statistically higher incidence of NEC. We did not detect NEC episodes in patients treated with CPX-351, 5 + 2 (cytarabine, idarubicine), and high-dose cytarabine. Thus, we found that cytarabine could determine mucosal damage when associated with an anthracycline but not if delivered either alone or as dual-drug liposomal encapsulation of daunorubicin/cytarabine. We also describe NEC mortality, symptoms at diagnosis, intestinal sites involvement, and prognostic significance of bowel wall thickening.

## 1 Introduction

For several decades, cytarabine plus an anthracycline (e.g., “7 + 3” regimen) has been the standard-of-care induction therapy for acute myeloid leukemia (AML), including therapy related AML (t-AML) and AML with myelodysplasia-related changes (AML-MRC) (1, 2). Although complete remission was historically achieved in 60–80% of patients aged < 60 years with AML, conventional chemotherapy may not be well tolerated either in older patients or in patients with significant comorbidities (3, 4). The key point of intensive induction treatment is the achievement of a complete response (CR) and furthermore may be a bridge to allogenic transplant (HCT) when indicated (5). CPX-351 is a dual-drug liposomal encapsulation of daunorubicin/cytarabine. CPX-351 has been approved for newly diagnosed t-AML and MRC-AML adult patients in US in 2017 and for patients aged ≥1 year in 2021, and it has been approved in EU/UK in 2018 (5–12). A major concern worldwide is the colonization of the intestine by multi-drug resistant bacteria (MDR) (13–15). Intensive induction and consolidation chemotherapy may damage intestinal mucosa leading to a neutropenic enterocolitis (NEC), a life-threatening complication with reported mortality up to 30-60%, although it has been recently suggested that an early US-guided diagnosis and prompt treatment may substantially improve the survival (16–19). NEC is clinical syndrome, and a bowel wall thickening (BWT), determined either by computed tomography (CT) or ultrasonography (US), has been proposed as a major diagnostic criterion (20–23). Different CHT regimens may be associated with different incidence of NEC (16). NEC incidence greatly varies from 0.8% to 46%. This variability is partly due to the retrospective nature of most studies, and partly due to different definitions and diagnostic criteria (23, 24). The aim of our study has been to investigate the incidence of NEC, symptoms at diagnosis, and outcome in AML patients exposed to different CHT regimens with respect to their potential damaging effects on intestinal mucosa. The novelty of our study relies on the homogenous cohort of patients (all affected by AML) and the prospective enrollment design.

## 2 Materials and methods

### 2.1 Patients and study design

This prospective study was conducted from March 2007 through March 2023 in the adult (>18 years old) Leukemia ward of the Hematology Unit at the University of Pisa (Italy). The patient population included all patients with newly diagnosed AML undergoing intensive chemotherapy and experiencing chemotherapy-related neutropenia (CHTN) (25–27). Overall, we enrolled N=213 patients. A single patient could receive multiple chemotherapy regimens during subsequent admissions on the ward. Each admission inducing a chemotherapy-related neutropenia episode (CHTNe) was considered a statistical “observation”, and each “observational period” started on the date of admission and ended on the date of discharge. Thus, in the N=213 patients we recorded N=465 CHTNe. A single patient could experience multiple “events” of NEC diagnosed during different observational periods. All the other AML patients who experienced chemotherapy-related neutropenia but did not experience NEC episodes (NECe) during an entire observational period were considered as the control group (N = 423 NEC negative observations). All patients provided written informed consent and the study was approved by our Institutional Review Board (IRB File 3636).

### 2.2 Definition of neutropenic enterocolitis

NEC was defined when BWT was found more than 4 mm in transversal scans for at least 30 mm length (28), at the onset of at least one of the following symptoms: fever (axillary temperature ≥38°C, (F)) and/or abdominal pain (AP) and/or diarrhea (more than three fluid stools/24 h, D) during neutropenia (23, 28, 29), which was defined as absolute neutrophil count (ANC) < 0.5 x 10^9^/L. Abdominal pain was evaluated using a Visual Analogous Scale Pain Score, ranging from 0 to 10 (28). Resolution of NEC was defined as a complete disappearance of symptoms combined with “restitutio ad integrum” of all bowel segments involved at diagnosis by bedside ultrasound (30).

### 2.3 Antimicrobial prophylaxis

From the start of the study until December 2013, all patients received levofloxacin 500 mg/day, fluconazole 400 mg/day, and aciclovir 400 mg twice a day until neutrophil recovery. In January 2014, levofloxacin and fluconazole prophylaxes were discontinued and patients received posaconazole prophylaxis (31).

### 2.4 Microbiological evaluation

**Figure 1:**
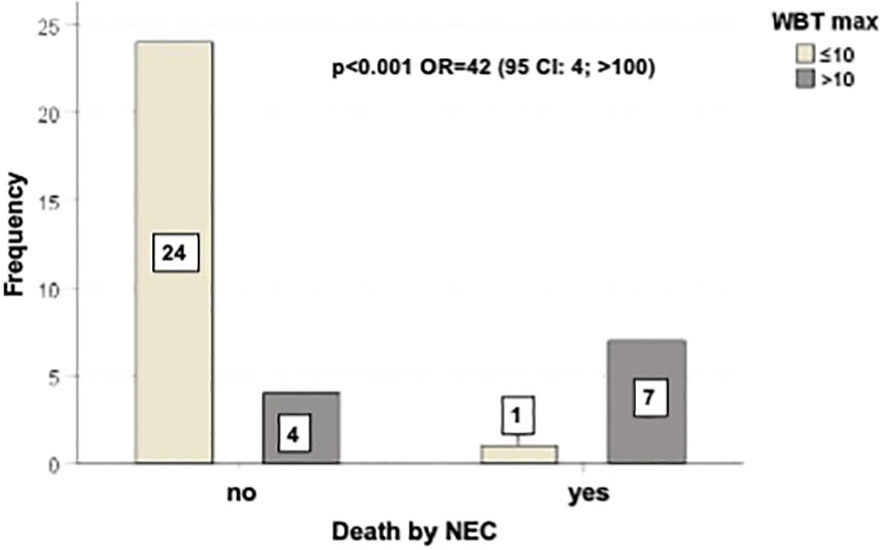
Outcome of patients correlated with BWT < 10 mm or ≥ 10 mm. There was a statistically significant difference in mortality rate between the two groups of patients (p=0.001, with OR=42).

Blood cultures were part of routine fever workup for all febrile episodes as per institutional policy as previously described (21, 32). Routine stool cultures were performed at each episode of diarrhea to rule out Clostridium Difficile colitis (28), or other bacterial or fungal infections. PCR analysis was carried out to rule out viral infections (herpes viruses, adenovirus, EBV, CMV, rotavirus, norovirus enterovirus, and astrovirus). Stool cultures were repeated if diarrhea persisted or worsened. Moreover, diarrhea was considered chemotherapy-induced (33) (**Figure 1**) when no pathogen was isolated from stool cultures, and neutropenic fever was considered of unknown origin if no infection was microbiologically documented using extensive microbiological evaluation (28, 32, 34).

### 2.5 Ultrasonographic examination

**Figure 2:**
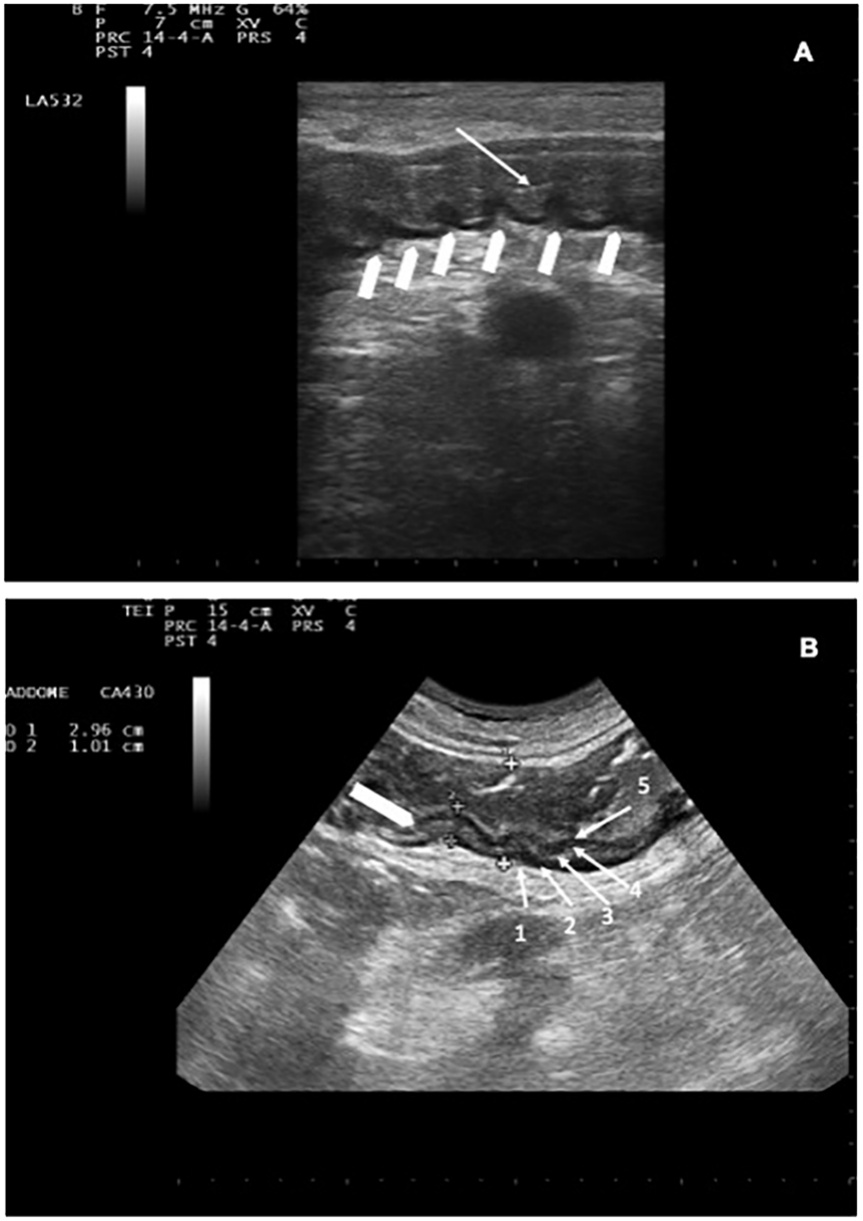
**(A)** Descending colon with BWT. White arrow indicates the lumen; white arrowheads indicate preserved haustra. **(B)** NEC involving the descending colon: BWT 10.1 mm (white arrowhead). Haustra are poorly recognizable. The whole colon measures 29.6 mm. The 5 layers are indicated by white arrows (1 = serosa, 2= muscolaris mucosa, 3=submucosa, 4=mucosa, 5= mucosa-lumen interface).

Each patient enrolled in the study underwent a baseline abdominal and intestinal B-mode US at the beginning of each observational period, as soon as admitted on the ward, before receiving any chemotherapy (16, 28, 32, 35). All patients were clinically monitored during their entire hospital stay (observational period). Ultrasound was performed with an Esaote MyLab 25 ultrasonographer equipped with a 3.5–5.0-MHz convex probe and a 7.5-MHz linear transducer without any preparation, 6–12 h within the onset of either one symptom or a combination of symptoms (fever and/or diarrhea and or abdominal pain). The entire gastrointestinal tract was submitted to a gray-scale US examination as previously described (35–37). The colon and ileum wall thickness were measured from the luminal surface to outer wall, as previously reported (16, 32, 34, 38, 39). Bowel wall layers were investigated, including superficial mucosal interface, deep mucosa, submucosa, muscolaris propria and serosa (40, 41) (**Figure 2A**).

The degree of dilation (42) and motility (43) were also assessed. The presence of haustra (**Figure 2B**) or dehaustration and the presence/absence of free abdominal fluid in all four quadrants and/or abdominal organ pathologies (such as cholecystitis or hepatolienal candidiasis) (23, 28) were assessed during each imaging study (36, 42, 43). Bowel wall was defined thickened if 4 mm in at least 3 cm long segments was found in transvers scans (19, 21, 28, 44), and bowel content was defined as gas, foodstuff or feces, mixtures of the two, or fluid filled (45). Patients diagnosed with NEC were considered our study group. Follow-up ultrasound was invariably repeated if clinical conditions worsened or at the onset of new symptom/s.

Asymptomatic patients received another bed side US to assess the gastrointestinal tract after five days of neutropenia. If they did not experience NEC episodes during the entire observational period, they were considered controls. Ultrasound studies were performed either on weekdays or during the weekend as clinically indicated. In all patients, bed-side US was performed by a hematologist member and teacher at the Italian School for Basic and Emergency Ultrasound (SIUMB) at the University of Pisa, with expertise in gastrointestinal ultrasound.

### 2.6 NEC treatment

After the ultrasonographic diagnosis of NEC, blood cultures were obtained in febrile patients, and a conservative approach with broad-spectrum antibiotics covering both Gram positive and -negative pathogens, anaerobes, and fungi, was immediately started regardless of symptoms. Treatment included meropenem, vancomicin, liposomal ampho-B; dosage was adjusted to renal function and caspofungin was used in patients with renal impairment and electrolyte imbalance. In sepsis/septic shock, IgM-enriched immunoglobulins were infused over three days (46, 47). Treatment was modified if infections were documented according to sensitivity tests. Patients also received total parenteral nutrition, G-CSF, fluid resuscitation, transfusions of packed red blood cells, platelet and fresh frozen plasma as needed (32). NEC-related mortality was defined according to manuscripts previously published (17, 18, 23, 32, 34, 39, 45, 48).

### 2.7 Statistical analysis

Categorical data were described by absolute and relative (%) frequency, continuous data were summarized by mean and standard deviation. To compare categorical factors with continuous and categorical variables t-test for independent samples and chi square test or z-test for two proportions were performed, respectively. Significance was fixed at 0.05 and all analyzes were carried out with SPSS v.28 technology.

## 3 Results

### 3.1 Characteristics

From March 2007 to March 2023, N=213 patients were prospectively enrolled in the study. Patients’ characteristics were the following: median age 52 years (range 19-81 years), females N= 98 (46.4%) and males N=113 (53.6%); median BMI 24.4 (range 14.1-42.6), N= 131 patients (61.5%) had BMI <25 and N= 82 patients (38.5%) had BMI ≥ 25, respectively. Overall, N=40/213 patients (18.7%) were acute myeloid leukemia (AML) with myelodysplasia-related changes (MRC-AML), and N= 13/213 patients (6%) were therapy-related AML (t-AML) (49).

### 3.2 Incidence of NEC

We diagnosed N=42 NECe out of N=465 chemotherapy related neutropenic observations (Ne) with an incidence rate of 9.0%. Thirty-six patients experienced N=1 episode of NEC, and N= 6 patients experienced 2 episodes of NEC. NEC was diagnosed after a median of N=4.5 days of neutropenia grade IV (range 1-12 days). There was not a statically significant difference between NECe occurring before and after antimicrobial prophylaxis change (before and after 2014, paragraph 2.3, p=0.434). We found that there was not a statistically significant impact of neutropenia on cumulative incidence (CI)-rate of NEC (p=0.279) (**Table 1**).

**Table 1:** Cumulative incidence rate percentage of NEC.

Two-year period	NEC no	NEC yes	Total	NEC percentage	Cumulative percentage of NEC yes (n=36)
2007-2008	23	2	25	8	5,6
2009-2010	20	5	25	20	19,4
2011-2012	22	6	28	21,4	36,1
2013-2014	18	2	20	10	41,7
2015-2016	26	9	35	25,7	66,7
2017-2018	28	4	32	12,5	77,8
2019-2020	23	3	26	11,5	86,1
2021-2022	17	5	22	22,7	100,0
Total	177	36	213	16,9	

The CI was performed only on patients experiencing 1 episode of NEC.

### 3.3 Number of cycles of chemotherapy and incidence of NEC

The number, the type of cycles of CHT, the number of NECe, and median length of days of neutropenia grade IV are reported in **Table 2**. We report the CHT regimens according to various protocols with antileukemic activity used in our leukemia unit during the study period (2007-2023). **Table 3** shows the patient clinical variables at the first and second episode of NEC. Microbiology-documented NEC infections (blood and/or stool) were found in N=31 out of 42 NECe (74%), 27 positive blood cultures and 4 positive stool cultures. In N= 23 out of 27 (85%) NECe we found positive blood cultures (N=9 Pseudomonas aeruginosa, and among these 4 were aminoglycosides, quinolones, and β-lactams resistant, respectively (57); N=3 Klebsiella pneumoniae carbapenemase (KPC)-producing bacteria; N=11 Escherichia coli, and among these 6 β-Lactamase (ESBL) were resistant); N=2 Enterococcus Faecalis; and N=2 Candida albicans. In 4 out of 27 NECe the stool cultures yielded pathogens (N=2 Candida albicans, and N=2 KPC, respectively).

**Table 2:** Number, type of cycles of CHT, number of NECe, and median length of days of neutropenia grade IV in AML patients during the study period (2007-2023).

Therapy	Total number of cycles delivered (n)	Median length neutropenia grade IV (range)	NECe number	NECe/Total (%)
5 + 2 (ida)	66	5 (2-13)	0	0
5 + 3+3 (cyta-ida-eto)	110	7 (1-22)	6	5.4
7 + 3 (dauno)	40	14 (6-22)	10	25
7 + 3 (ida)	120	13 (8-22)	17	14.2
AML 1310 (ind)	17	14 (5-24)	8	47
AML 1310 (cons)	11	7 (5-14)	1	9.1
HD ARA-C	64	2 (0-6)	0	0
CPX-351 (ind1)	22	19.5 (12-24)	0	0
CPX-351 (ind2)	1	9 (NA)	0	0
CPX-351 (cons)	14	11 (6-22)	0	0
*Tot*	465		42	9.0

5 + 2 (ida)= idarubicin 13 mg/m^2^/d for 2 days, and cytarabine 100 mg/m^2^/d as a continuous for 5 days (50)); 5 + 3 + 3 (cyta-ida-eto); cytosine arabinoside (100 mg/m^2^, six doses), idarubicin (8 mg/m^2^, three doses), and etoposide (100 mg/m^2^, five doses) (51); 7 + 3 = 7-day continuous infusion of cytarabine at the dosage of 200 mg/m^2^ per day on days 1 to 7 and daunorubicin at 60 mg/m^2^ per day on days 1 to 3 (52); 7 + 3 ida = idarubicin 12 mg/m^2^ for 3 days and cytarabine was given in a dose of 100 mg/m^2^ continuous IV infusion for 7 days (53); GIMEMA AML1310 (ind)= induction consisted of IV daunorubicin 50 mg/m^2^ daily on days 1, 3, and 5; IV etoposide 50 mg/m^2^ daily on days 1 to 5; and IV cytarabine 100 mg/m^2^ as a daily continuous infusion, days 1 to 10 (54); GIMEMA AML1310 (cons) = IV daunorubicin 50 mg/m^2^ daily on days 4, 5, and 6 and IV cytarabine 500 mg/m^2^ every 12 hours on days 1 to 6 (54); HD ARA-C: consolidation with high dose cytarabine 3g/m^2^ every 12 h on days 1,3,5 (55, 56). CPX-351 ind1: CPX-351 100 units per m² (daunorubicin 44 mg/m² plus cytarabine 100 mg/m²) as a 90-min infusion on days 1, 3, and 5 (57); CPX-351 ind2: CPX-351 100 units per m² (daunorubicin 44 mg/m² plus cytarabine 100 mg/m²) as a 90-min infusion on days 1, 3 (57); CPX-351 cons: 65 units per m² (daunorubicin 29 mg/m² plus cytarabine 65 mg/m²) administered as a 90-min infusion on days 1 and 3 (57).

**Table 3:** Patient clinical variables at the first and second episode of NEC.

	First NEC episode		Second NEC episode	
Characteristic	Frequency/Mean	%/sd	Frequency/Mean	%/sd
Small intestine				
No	29	80.5	6	100
Yes	7	19.5	0	0
Colon				
No	12	33.3	1	16.7
Yes	24	66.7	5	83.3
Small intestine +Colon				
No	31	86.1	5	83.3
Yes	5	13.9	1	16.7
Fever at diagnosis				
No	36	100	6	100
Yes	0	0	0	0
Diarrhea				
No	36	100	6	100
yes	0	0	0	0
Pain				
No	33	91.7	5	84
Yes	3	8.3	1	16
Fever + Diarrhea				
No	16	44.5	2	33.3
yes	20	55.5	4	66.7
Fever + Abdominal Pain				
No	11	30.6	1	16.7
Yes	25	69.4	5	83.3
Diarrhea + Abdominal Pain				
No	11	30.6	2	33.3
Yes	25	69.4	4	66.7
Fever + Diarrhea + Abdominal Pain				
No	17	47.2	2	33.3
yes	19	52.8	4	66.7
Documented infections				
No	13	36.0	2	33.3
Yes	23	64.0	4	66.7
Therapy				
Surgical	3	8.3	0	0
Medical	33	91.7	6	100
Outcome				
deceased	7	19.4	1	16.7
alive	29	80.6	5	83.3
Chemotherapy				
3 + 3+5	3	8.3	3	50
3 + 7 IDA	16	44.5	1	16.7
3 + 7dauno	9	25.0	1	16.7
AML1310 ind	8	22.2	0	0
AML1310 cons	0	0	1	16.7
**WBT (mm)**	8.9 (mean value)	3.57	10.9	2.7
**Time to death (hours)**	53.3 (mean value)	22.2	0	0
**Time to surgery (hours)**	26.7 (mean value)	18.9	0	0

Gram-negative bacteria in blood cultures were the most represented 85% (N= 23/27), 56% were MDR bacteria, and patients colonized were 43.5% (N=10/23). There was not a statically significant impact of colonization on NEC episodes in the chemotherapy sub-groups in which NEC was diagnosed: in the 5 + 3 + 3 (cyta-ida-eo) (p= 0.884), in the 7 + 3 (dauno) (p=0.412), in the 7 + 3(ida) (p=0.732), in the AML 1310(ind) (p=0.132) and in the AML 1310 (cons) (p=0.836). In the group of patients treated with CPX-351(ind1) N=2 patients, and CPX (cons) N=2 patients were colonized by Klebsiella pneumoniae carbapenemase (KPC)-producing bacteria, and none experienced a NECe.

Overall, AML 1310 (ind), followed by 7 + 3 (dauno), 7 + 3 (Ida), AML 1310 (cons), and 5 + 3 + 3 were the chemotherapy cycles most associated with occurrence of NECe. **Table 4** reports the statistical comparison of two cycle of chemotherapy which had the highest percentages of NECe used as reference with respect to other cycles. In column 4 of **Table 4**, AML 1310 (ind) is the chemotherapy used as reference with the highest incidence of NEC and it was compared with NECe occurring using the other cycles of chemotherapy. In column 5 of **Table 4**, 7 + 3 (dauno) is considered the reference chemotherapy. The comparison of CI-rate incidence of NEC in patients treated with CPX-351 (ind1) *vs* 7 + 3 (ida) showed a strong trend (p=0.06). We did not find a statistically significant difference in CI-rate incidence of NEC between 7 + 3 using daunorubicin or idarubicin (p=0.115). In univariate analysis the only variable associated with NEC occurrence was the type of chemotherapy (**Table 4**). Neutropenia, patients’ age, year of treatment, did not have a statically significant impact (p= p=0.279, 0.292, p=0.434, respectively).

**Table 4:** Comparison of NECe related to chemotherapy cycles.

Therapy	Total number	NEC/Total (%)	p-value	p-value
5 + 2 (ida)	66	0	<0.001	<0.001
5 + 3+3 (ida-eto-cyta)	110	5.4	<0.001	0.002
7 + 3 (dauno)	40	25	0.186	**Reference**
7 + 3 (ida)	120	14.2	0.003	0.182
AML 1310 (ind)	17	47	**Reference**	0.186
AML 1310 (cons)	11	9.1	0.092	0.471
HD ARA-C	64	0	<0.001	<0.001
CPX-351 (ind1)	22	0	0.001	0.028
CPX-351 (ind2)	1	0	NA	NA
CPX-351 (cons)	14	0	0.010	0.094
*Tot*	465	9.0		

5 + 2 (ida)= idarubicin 13 mg/m2/d for 2 days, and cytarabine 100 mg/m2/d as a continuous for 5 days (50); 5 + 3 + 3 (ida-eto-cyta); idarubicin (8 mg/m2, three doses), cytosine arabinoside (100 mg/m2, six doses) and etoposide (100 mg/m2, five doses) (50); 7 + 3 = 7-day continuous infusion of cytarabine at the dosage of 200 mg/m2 per day on days 1 to 7 and daunorubicin at 60 mg/m2 per day on days 1 to 3 (50); 7 + 3 ida = idarubicin 12 mg/m2 for 3 days and Cytarabine was given in a dose of 100 mg/m2 continuous IV infusion for 7 days (53); GIMEMA AML1310 (ind)= Induction consisted of IV daunorubicin 50 mg/m2 daily on days 1, 3, and 5; IV etoposide 50 mg/m2 daily on days 1 to 5; and IV cytarabine 100 mg/m2 as a daily continuous infusion, days 1 to 10 (53); GIMEMA AML1310 (cons) = IV daunorubicin 50 mg/m2 daily on days 4, 5, and 6 and IV cytarabine 500 mg/m2 every 12 hours on days 1 to 6 (53); HD ARA-C: consolidation with high dose cytarabine 3g/m^2^ every 12h on days 1,3,5 (55, 56); CPX-351 ind1: CPX-351 100 units per m² (daunorubicin 44 mg/m² plus cytarabine 100 mg/m²) as a 90-min infusion on days 1, 3, and 5 (57); CPX-351 ind2: CPX-351 100 units per m² (daunorubicin 44 mg/m² plus cytarabine 100 mg/m²) as a 90-min infusion on days 1, 3 (57); CPX-351 cons: 65 units per m² (daunorubicin 29 mg/m² plus cytarabine 65 mg/m²) administered as a 90-min infusion on days 1 and 3 (57).

### 3.4 Mortality

Eight patients died (N=8/42, 22%) because of a NEC-related septic shock. Seven out of 8 died during the first NECe, and one died during the second NECe. The mortality rate of patients who experienced only one NECe was 19.4% (7/36) and in patients experiencing 2 NECe was 16.7% (1/6), without a statistically significant difference (p = 0.877).

### 3.5 Treatment

Thirty-nine NECe were treated conservatively whereas N= 3 NECe were treated with surgery. Six out of 39 patients and two out of three patients died because of uncontrolled septic shock in the group treated with conservative therapy and surgery, respectively, without a statistically significant difference between the two groups (p=0.165).

### 3.6 Bowel wall thickness

**Figure 3:**
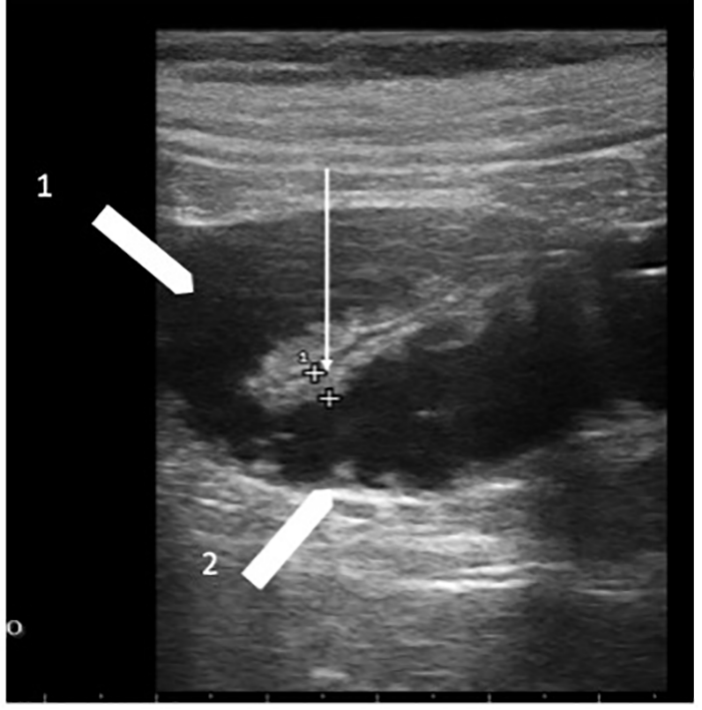
Chemotherapy-related diarrhea. Two ileum loops with bowel wall measuring 3.0 mm (white arrow); white arrowhead 1 indicates the lumen liquid-filled; white arrowhead 2 indicates valvulae conniventes.

**Figure 3** shows the outcome of patients correlated with BWT < 10 mm or ≥ 10 mm. There was a statistically significant difference in mortality rate between the two groups of patients (p=0.001, with OR=42).

### 3.7 Body mass index

Body mass index (BMI) did not influence BUS scanning of patients enrolled in the study. Mean BMI of the entire NEC group was 24.3 ± 4.3; mean BMI of the NEC negative control group was 25 ± 5.2. Patient’s BMI was homogeneously distributed in the two groups (p=0.397).

### 3.8 Bowel sites involved in NEC

Intestinal involvement in NECe was as follows: N=7/42 (16.7%), N=29/42 (69%), and N=6/42 (14.3%) had small bowel, colon, and both involved, respectively. Thus, the colon (alone and in association with the small bowel) was the most involved site. One and seven patients died when NEC was localized in the small bowel and colon, respectively.

### 3.9 Symptoms

Symptoms at NECe diagnosis are listed in **Table 3**. At NECe diagnosis fever and diarrhea alone were never found, whereas abdominal pain alone was found in 8.3% and in 16% of patients who experienced one and two NECe, respectively. The most frequent symptom or symptoms at NECe diagnosis were fever + abdominal pain, followed by diarrhea + abdominal pain, fever + diarrhea, and fever+ diarrhea + abdominal pain.

## 4 Discussion

Chemotherapy agents used to treat patients affected by AML may cause direct intestinal injury leading to NEC (16–18, 23, 28, 45). Moreover, an emerging concern is the colonization of the intestine by MDR bacteria in patient who will receive intensive CHT (13).

A part of patients diagnosed with AML will be offered an allogenic transplant (1, 2) after remission-induction CHT, and in these patients the colonization by MDR bacteria may be detrimental (1, 2). In our research we wanted to verify if different CHT regimens could cause a significantly different mucosal injury causing a different incidence of NEC.

### 4.1 Pathogenesis

The exact pathogenesis of NEC is multi-factorial (16). The cytotoxic effect of chemotherapy can cause direct mucosal injury and destruction of the normal mucosal architecture (17, 24, 38), with loss of gut barrier function and subsequent microbial invasion of the bowel wall by colonic and opportunistic organisms (58). Bacterial endotoxins may lead to inflammation, edema, ulceration, transmural necrosis, and perforation. Intramural hemorrhage (17) due to severe thrombocytopenia (20, 24) may lead to massive gastrointestinal (GI) bleeding. Neutropenia reduces the immune response against the microbial invasion of the bowel wall (20, 24) and neutropenia alone can cause mucosal ulcerations (16, 20). Complications of NEC are perforation of the bowel wall, stenosis, fistula, ileus or sub-ileus, gastro intestinal bleeding, and septicemia with or without septic shock (ref (16). and references therein).

### 4.2 Incidence

In a systematic review of 21 studies, the reported pooled incidence rate of NEC was 5.3% (266/5058; 95% CI, range 4.7%–5.9%) in adult patients hospitalized for the treatment of hematologic malignancies, high-dose chemotherapy in solid tumors, or aplastic anemia (23). In our prospective study in AML patients, CI-rate incidence of NEC was 9.0%.

Incidence and outcome of NEC occurring in patients affected by acute leukemia undergoing CHT were reported previously in one prospective study (28) and two retrospective studies (16, 45). Gorshluter et al. in their study included 36 patients with acute leukaemia (AML, acute lymphoblastic leukaemia (ALL) or blast crisis of chronic myeloid leukaemia (CML) with 62 independent episodes of neutropenia after myelosuppressive chemotherapy and reported a NEC incidence of 6.5% (28). Cartoni et al. reported overall 6% NEC incidence in N=64 patients with AML (72.8%), N=21 patients (23.8%) with ALL, and N=3 patients (3.4%) with CML in blast crisis (45). Pugliese et al. reported overall 23.8% NEC incidence in N=420 patients with AML (16). Unlike the prospective study of Goshluter et al. (28), our cohort included only patients diagnosed with AML. Our study also differs from the other two studies (16, 45) due to the prospective *vs* retrospective design. CI-rate incidence of NEC in our study was 9.0% NEC, diagnosed at a median of 4.5 day of neutropenia grade IV. Cytotoxic chemotherapeutic agents (i.e., cytosine arabinoside, vinca alkaloids, and doxorubicin) account for most cases of NEC, but other agents have been also implicated (e.g., 5-fluorouracil, gemcitabine, vincristine, methotrexate, leucovorin, and taxane-based agents) (59).

We found that the highest incidence of NEC occurred in patient treated with the following regimens: GIMEMA AML1310 (ind) (47%), followed by 7 + 3 (dauno) (25%), which were used as reference for statistical comparison with the other CHT cycles, followed by 7 + 3 (ida), AML1310 (cons), and 5 + 3 + 3 (**Table 4**). Our findings are in accordance with what previously reported by Pugliese et al. (16), who analyzed the impact on intestinal mucosal toxicity of the various schedules of cytarabine-based cytotoxic agents reporting that patients treated with high (AML-12 trial, and FLAG-Ida protocol), intermediate (HOVON-SAKK trial), and standard (AML-10 trial) dose cytarabine chemotherapy regimens experienced NEC with an incidence of 42%, 19%, and 15%, respectively. It was previously reported that neutropenia and not the underlying disease acute leukemia was the main risk factor for NEC (23). In our study we found that the median duration of neutropenia in patients treated with CPX-351 was about 25% longer in respect to the median of days of patients treated with 7 + 3 (dauno), and 7 + 3 (ida). Nevertheless, we did not find NEC episodes in patients treated with CPX-351 neither in induction (1 or 2) nor in consolidation. CPX-351 is a liposomal encapsulation of daunorubicin and cytarabine at a synergistic 1: 5 molar ratio (49). This formulation gave a statistically significant difference in CI-rate incidence of NEC if compared with a standard induction based on 7 days of cytarabine +3 days of daunorubicine (**Table 4**) and a strong trend if CPX-351 is compared with 7 + 3 (ida) (p=0.06). This observation might suggest that the damage of liposomal encapsulation of daunorubicin and cytarabine on the intestinal mucosa is minimum. Moreover, we might also speculate that the mucosal and submucosal injury caused by CHT, rather than the length of neutropenia, has a major impact on the possibility to develop bowel wall infection and NEC.

It was recently reported by Chiche et al. in a French retrospective multicenter study, that 50% of patients had all grade of gastro intestinal toxicity but only 4 AML patients out of 103 patients treated with CPX-351, experienced gastro intestinal toxicity grade 3 (vomiting in 1 patient a mucositis in 3 patients) (49). Lemoli et al. in their real-world experience with CPX-351 study found that gastro intestinal toxicity was rarely reported in real-world studies (5). Hueso et al. addressed the mechanism of mucosal damage from 7 + 3 with daunorubicin (60). Moreover, Renga et al. (submitted manuscript) fond that thigh junction protein of the mucosal epithelial barrier are preserved in cells exposed to CPX-351 but not if exposed to the 3 + 7 (dauno) combination (61). In our study NEC was diagnosed after a median of 4.5 days of neutropenia (range 1-12 days). Thus, NEC episodes were diagnosed relatively early in the neutropenic phase post chemotherapy. The median of days of neutropenia for each chemotherapy cycle is reported in **Table 2**. NEC episodes were not diagnosed after 12 days of neutropenia (range 1-12 days) although neutropenia related to chemotherapy could exceed 12 days. We did not detect delayed NEC episodes after 12 days although neutropenia may persist much longer (for example in the CPX-351 (ind) the neutropenia phase ranged from 12 to 24 day, as in AML1310 (ind)). Thus, NEC seems a relatively early event post end of chemotherapy not happening later even though neutropenia persists. Thus, considering the literature and our findings, it is reasonable to interpret the results reported by the authors (5, 49) hypothesizing that in this setting of patients the mechanism of mucosal barrier damage is most probably more due to a direct effect of the chemo-agents delivered rather than on the neutropenia *per se*. Furthermore, the impact of chemotherapy on mucosal barrier damage, is part of the pathogenesis addressed by the literature (16, 17, 20, 24, 38, 58). In accordance with the literature mentioned (5, 49) we found a correlation of NEC with the type of chemotherapy and not with the duration of neutropenia. Other CHT regimens in which we did not detect NEC episodes were HD-ARAC (55, 56), and 2 + 5 (ida) (50). Thus, cytarabine may damage intestinal mucosal and submucosal architecture only if combined with daunorubicin or idarubicin and does not induce mucosal injury at either reduced combination schemes (e.i. 2 + 5 ida) or if combined as a dual-drug liposomal encapsulation with daunorubicin (CPX-351).

### 4.3 Diagnosis and outcome

High-resolution ultrasound techniques allow detailed differential diagnosis including NEC (21, 28, 37, 62). We found that patient ‘s BMI did not affect the reliability of the US evaluation of the intestinal tract as previously reported (30, 32, 37, 42, 43), which allows to scan the intestine at bed-side in severely ill patients.

We found that the analysis of symptoms at NEC diagnosis in the setting of AML patients with chemo-related NEC agrees with data previously reported (32). In this study, focused on AML patients, we did not find neither fever nor diarrhea alone (**Table 3**). Abdominal pain was the symptom most frequently found either alone or in combination with other symptoms at diagnosis (**Table 3**). The mean time to death and surgery at NEC first episode was 53.3 hours and 26.7 hours, ant at second episode of NEC 22.2 hours and 18.9 hours, respectively. Thus, our study confirms the crucial benefit of an early US bed-side diagnosis and a timely treatment intervention (32).

Mortality of NEC can be up 30–60%, and is due to sepsis, uncontrolled bleeding, or necrotizing perforation (16, 23). Recently authors have shown that the mortality can be reduced significantly with an early US-driven diagnosis and prompt treatment (16, 17, 32). NEC-related mortality in the setting of patients affected by leukemia, previously reported by Gorshluter et al. (28), Pugliese et al. (16), and Cartoni et al. (45), was 50%, 23%, and 29%, respectively. In our study NEC-related mortality was 22% without a statistically significant difference (p= 0.308, p= 0.448, and p=0.207 respectively). Drug-resistant Gram-negative bacilli may contribute to significant morbidity and mortality in colonized patients undergoing chemotherapy (13). Accordingly, in our study 85% of documented infections were Gram-negative bacteria, and 56% of them were MDR which is indeed a public health problem of major concern worldwide. BWT is considered to establish the clinical diagnosis of NEC (20, 45), and it reflects the pathology of NEC. On macroscopic viewing (either surgical pathology, or on autopsy), there is transmural inflammation of the bowel (45), or transmural necrosis (17), which is edematous, dilated and thickened (45). Cartoni et al. reported in their retrospective study that the wall thickness had prognostic relevance with mortality rate of 60% in patients with BWT ≥ 10 mm (45).

In our prospective study we found that BWT ≥ 10 mm maintains its prognostic significance (**Figure 1**). Moreover, the results of our study further confirm that BWT is pathognomonic of NEC. None of the patients in the NEC-negative control group experienced BWT. This finding, previously observed in other studies (28, 32), is crucial in clinical practice to make NEC diagnosis at patient’s bedside, because patient clinical conditions and thrombocytopenia often preclude tissue biopsies given the high risk of bleeding and perforation (23). Clinical symptoms combined with BWT (determined either by ultrasonography (US) or computed tomography (CT) (20–22)) are considered highly suggestive diagnostic criteria (23, 32, 63), as proved by the revision of NEC diagnostic criteria (21, 24, 28, 32).

## 5 Conclusions

There is a growing concern of drug resistant bacteria colonizing the bowel of patients affected by AML. Different chemotherapy regimens show different mucosal and submucosal architecture damage of the intestinal tract leading to a significant difference in NEC incidence. Bed-side US is a non-invasive, radiation-free, and widely available imaging technique, which allows an early diagnosis of NEC with a timely intervention of a mucosal damage leading to NEC at bed side. Our findings have shown that various chemotherapy regimens determine different intestinal mucosal damage resulting in a significant different NEC incidence. These results may be valuable in planning the induction and consolidation treatment strategy, especially in patients colonized with drug resistant bacteria who might be candidate to receive an allogenic transplant post remission treatment. In these patients it is desirable to obtain a remission without experiencing life-threatening complications that could preclude further therapeutic options inducing life-threatening chemotherapy-related complications.

## Data availability statement

The raw data supporting the conclusions of this article will be made available by the authors, without undue reservation.

## Ethics statement

The studies involving humans were approved by Comitato Etico Area Vasta Nord Ovest (Tuscany). The studies were conducted in accordance with the local legislation and institutional requirements. The participants provided their written informed consent to participate in this study.

## Author contributions

EBe: Conceptualization, Data curation, Investigation, Methodology, Supervision, Writing – original draft, Writing – review & editing, Validation. GT: Data curation, Methodology, Writing – review & editing. GP: Data curation, Writing – review & editing. RM: Data curation, Formal Analysis, Methodology, Validation, Writing – original draft. EBr: Methodology, Validation, Writing – original draft, Writing – review & editing. PL: Methodology, Writing – review & editing. MS: Data curation, Investigation, Writing – review & editing. EM: Data curation, Writing – review & editing. RG: Methodology, Writing – review & editing. FM: Investigation, Methodology, Writing – review & editing. EC: Conceptualization, Methodology, Supervision, Writing – review & editing. EN: Supervision, Writing – review & editing. CA: Data curation, Investigation, Methodology, Writing – review & editing. FC: Investigation, Methodology, Writing – review & editing. LS: Supervision, Writing – review & editing. KV: Investigation, Methodology, Writing – review & editing. SS: Methodology, Writing – review & editing, Investigation, Supervision. VR: Methodology, Validation, Writing – review & editing. BB: Supervision, Validation, Writing – review & editing, Methodology. SG: Writing – review & editing, Supervision, Validation.
